# Drosophila KDEL Receptor Function in the Embryonic Salivary Gland and Epidermis

**DOI:** 10.1371/journal.pone.0077618

**Published:** 2013-10-18

**Authors:** Elliott W. Abrams, Yim Ling Cheng, Deborah J. Andrew

**Affiliations:** Department of Cell Biology, the Johns Hopkins University School of Medicine, Baltimore, Maryland, United States of America; University of Massachusetts Medical School, United States of America

## Abstract

Core components of the secretory pathway have largely been identified and studied in single cell systems such as the budding yeast *S. cerevisiae* or in mammalian tissue culture. These studies provide details on the molecular functions of the secretory machinery; they fail, however, to provide insight into the role of these proteins in the context of specialized organs of higher eukaryotes. Here, we identify and characterize the first loss-of-function mutations in a KDEL receptor gene from higher eukaryotes. Transcripts from the Drosophila KDEL receptor gene *KdelR* – formerly known as *dmErd2* – are provided maternally and, at later stages, are at elevated levels in several embryonic cell types, including the salivary gland secretory cells, the fat body and the epidermis. We show that, unlike *Saccharomyces cerevisiae Erd2* mutants, which are viable, *KdelR* mutations are early larval lethal, with homozygous mutant animals dying as first instar larvae. *KdelR* mutants have larval cuticle defects similar to those observed with loss-of-function mutations in other core secretory pathway genes and with mutations in *CrebA*, which encodes a bZip transcription factor that coordinately upregulates secretory pathway component genes in specialized secretory cell types. Using the salivary gland, we demonstrate a requirement for *KdelR* in maintaining the ER pool of a subset of soluble resident ER proteins. These studies underscore the utility of the Drosophila salivary gland as a unique system for studying the molecular machinery of the secretory pathway *in vivo* in a complex eukaryote.

## Introduction

 The rough endoplasmic reticulum (ER) is where synthesis of secreted and transmembrane proteins occurs through the process of cotranslational translocation. The lumen of the ER – the first compartment of the secretory organellar network – provides a selective environment for the folding and primary modifications of both secreted and transmembrane proteins. Thus, the ER contains many soluble resident proteins, such as chaperones, which facilitate the translocation and correct folding of nascent polypeptides (reviewed in 1). Soluble resident proteins are maintained in the ER through a retrieval system involving a carboxy-terminal signal and a transmembrane receptor that binds to that signal. In yeast, soluble ER resident proteins contain a four-residue carboxy-terminal Histidine-Aspartate-Glutamate-Leucine – HDEL – motif [2,3], which interacts directly with the seven transmembrane protein encoded by the *Erd2* gene [4]. In mammalian systems, a Lysine-Aspartate-Glutamate-Leucine – KDEL – motif or some variant of KDEL, is both necessary and sufficient for the retrieval of soluble ER proteins. The requirement for the KDEL motif was demonstrated by removing it from a lumenal ER-resident protein expressed in COS cells and showing that the protein was secreted into the media [5]. The sufficiency of the C-terminal motif was demonstrated in experiments in which non-ER proteins were redirected to the ER simply by the addition of KDEL sequences to their C-termini and by showing that mutations in the KDEL sequences in these chimeric proteins resulted in a loss of ER localization [6]. Increasing KDEL receptor levels also affects ER localization; cells overexpressing the receptor have increased retention even of proteins with very weak retention signals [7]. These experiments clearly demonstrate the importance of the KDEL receptor system in ER retrieval. Nonetheless, a genetic loss-of-function model equivalent to that in yeast has been lacking in higher eukaryotes. 

 A number of studies have demonstrated that different organisms can potentially utilize ER retention motifs from other species. Studies comparing the yeasts *S. cerevisiae* and *K. lactis*, which utilizes a DDEL terminal motif, have shown that although *S. cerevisiae* is only capable of retrieving HDEL and not DDEL proteins, *K. lactis* can retrieve proteins with both types of retrieval signals [8]. In addition, although there are a very few examples of endogenous mammalian proteins with HDEL motifs, an HDEL sequence can function in mammalian ER retrieval as shown in experiments with SEHDEL fused to the C-terminus of lysozyme [9]. However, despite high levels of similarity in overall structure, the human KDEL Receptor 1 cannot functionally replace the *S. cerevisiae* gene [10]. Plants are thought to use both KDEL and HDEL retrieval signals [11], which may also be the case with Drosophila ER resident proteins. A survey of the Drosophila proteome suggests that there are proportionally similar numbers of proteins containing C-terminal HDEL and KDEL motifs ([12].

 The Drosophila salivary gland is a useful system for studying the cell biology of secretion in an intact organ *in vivo*. A number of secretory pathway components are expressed to very high levels in the embryonic salivary gland, consistent with the specialization of the salivary gland for secretion [13,14]. The relatively large secretory cells of the salivary gland facilitate localization studies of secretory proteins and organelles (RM Fox and DJA, unpub.). The high level of secretion that occurs in the late embryonic salivary gland allows for direct immunohistochemical analysis of secretory function. Importantly, there is typically only a single Drosophila gene encoding each core secretory pathway component, whereas in vertebrates there are often multiple genes encoding the same or highly related proteins, complicating analysis of gene function due to functional redundancy. Thus, there is enormous potential for studying genetic interactions among secretory pathway component genes using the Drosophila salivary gland as a model system. 

Here, we describe the embryonic expression pattern of the Drosophila *KdelR* and demonstrate that loss-of-function mutations in the gene lead to defects in the cuticle secreted by the epidermal cells. We also show that KdelR function is required to maintain the pool of a subset of soluble endoplasmic reticulum (ER) resident proteins. Although this function for *KdelR* is expected based on work in other systems, our studies reveal that not all soluble ER residents share the same requirement for this receptor *in vivo*. Our work demonstrates the utility of the Drosophila salivary gland as an *in vivo* system for studying the secretory pathway, potentially revealing similarities and differences among biological systems.

## Methods

### Sequencing and protein alignments

 The *KdelR* cDNA (CK00230) was identified by its up-regulation in the embryonic salivary gland through the expression database at the Berkeley Drosophila Genome Project (BDGP)[15]. To determine the molecular lesions in the EMS alleles of *KdelR*, genomic DNA was prepared from *31Em* ^*1*^ and *31Em* ^*2*^ heterozygous adult flies using standard procedures [16]. The *KdelR* ORF was amplified using the following primer pair: erd(5)seq-5’GTTCCGTGACGCAGCCGCAG and erd(3)seq-5’GTGAGTGCAGTTCGGAAAACGG. PCR products were purified using the Qiagen (Valencia, CA) gel purification protocol and both strands were sequenced at the Johns Hopkins Core Sequencing Facility. KDEL-R sequences of various organisms were aligned using the Clustal_W [17] and Boxshade programs available at the Biology Workbench Version 3.2 (http://seqtool.sdsc.edu/CGI/BW.cgi).

### Fly strains

The following deficiency stocks were used to map the *KdelR* gene: *Df*(*2L*)*J3*, *Df*(*2L*)*J16*, *Df*(*2L*)*J17*, *Df*(*2L*)*J27* and *Df*(*2L*) *J106* (Flybase; [18]). The following EMS mutant strains that map to cytological region 31E were a generous gift from the laboratory of T. Grigliatti: *l(2*)*31Ek*, *l(2*)*31El*, *l(2*)*31Em* ^*1*^, *l(2*)*31Em* ^*2*^ *, l(2*)*31Ep*, *l(2*)*31Eq*, *l(2*)*31er* [18]. The lethal P-element line, *l(2*)*k00311*, was obtained from the Bloomington Drosophila Stock Center. 

### *In situ* hybridization and antibody staining

*in situ* hybridization and antibody staining were performed as previously described [19,20]. Antibody dilutions used in this study are as follows: α-β-galactosidase (1:5000), α-PH4α-SG1 (for light microscopy-1:20,000, for confocal microscopy- 1:5,000), α-Boca (1:500) and α-Wbl (1:25). PH4α-SG1 is a polyclonal antiserum made in rat [21] and α-Boca is a polyclonal antiserum made in guinea pig [22]. α-Wbl is a mouse monoclonal antibody [23]. βtub-E7 is a mouse monoclonal antibody obtained from the Drosophila Hybridoma Studies Bank (DHSB; Iowa City, IA). Biotin-conjugated secondary antibodies were obtained from Vector Labs (Burlingame, CA) and were used at a dilution of 1:500. All fluorescent secondary antibodies were from Molecular Probes (Eugene, OR) and used at a dilution of 1:400. Confocal images were captured using an Ultraview Confocal Microscope (Perkin Elmer) at the Johns Hopkins Microscope Facility. All other images were taken on a Zeiss Axiophot microscope with a Nikon Coolpix 4500 digital camera.

### Expression and analysis of wild-type and mutant ER proteins in S2 cells

Full length and KDEL/KEEL deleted *boca* and *wbl* ORFs were PCR amplified from cDNAs SD08653 (*boca*) and IP02648 (*wbl*) using the LongAmp Taq PCR kit (NEB) and the primers in Table 1.

**Table 1:** Primers used for amplification and subcloning full length and KDEL/KEEL deleted versions of Boca and Windbeutel into an expression vector.

boca cacc 5 prime	caccATGCAAACACGTCTTGTTTTG
boca stop 3 prime	CTACAGTTCGTCCTTTTTCGC
boca del529-540 stop 3 prime	ctaTTTCGCATTGACACCTGGGTA
wbl cacc 5 prime	caccATGATGCATATTTTGGTGACTCTG
wbl stop 3 prime	TCACAGTTCCTCCTTTTCCGG
wbl del760-771 stop 3 prime	tcaTTCCGGCGCTGTCTTGGTGAC

Amplified PCR products were first cloned into pENTR/D-TOPO (Invitrogen) and subsequently into pAW Gateway vector (http://emb.carnegiescience.edu/labs/murphy/Gateway%20vectors.html) using the LR clonase II kit (Invitrogen). Each construct was verified by sequencing. S2 cells were tranfected in six-well plates with 400 ng DNA at 45 - 60% confluency following the QIAGEN Effectene Reagents protocol. Cells were subsequently grown for 48 hours at 25 °C. Based on immunostaining, 10-20% of cells were transfected. Cell and media were collected and centrifuged at 14,000 rpm for 10 min at 4 °C to separate the cell pellet and media supernatant. The cell pellet was washed and resuspended in 1X PBS and boiled with 2X sample buffer prior to loading. The media supernatant was TCA precipitated and the resulting protein pellet was mixed with 1X PBS and boiled with 2X sample buffer prior to loading. Samples were run on a 5% stacking gel / 12% resolving gel at 30 mA. Protein was transferred to methanol pretreated PVDF membrane O/N at 4 °C in transfer buffer (39 mM glycine, 48 mM Tris base, 0.037% SDS, 20% Methanol). For immunostaining, membranes were first blocked with 5% milk:PBS-Tween for 1hr at RT and subsequently incubated with primary antibodies (αBoca (guinea pig) 1:10,000; αWbl (mouse) 1:250 ; αβtub-E7 (mouse) 1:1000) in 5% milk:PBS-Tween overnight at 4 °C. Membranes were washed and incubated with secondary antibody (goat αguinea pig HRP or goat αmouse HRP, both at 1:10,000) in PBS-Tween for two 2 hr at room temperature. Membranes were washed and HRP signal was subsequently detected using the Novex ECL HRP Chemiluminescent Substrate Reagent Kit (Invitrogen). These experiments were done twice.

## Results

### *Drosophila* KDELR has high sequence similarity to mammalian, *C. elegans* and *S. cerevisiae* homologs

**Figure 1:**
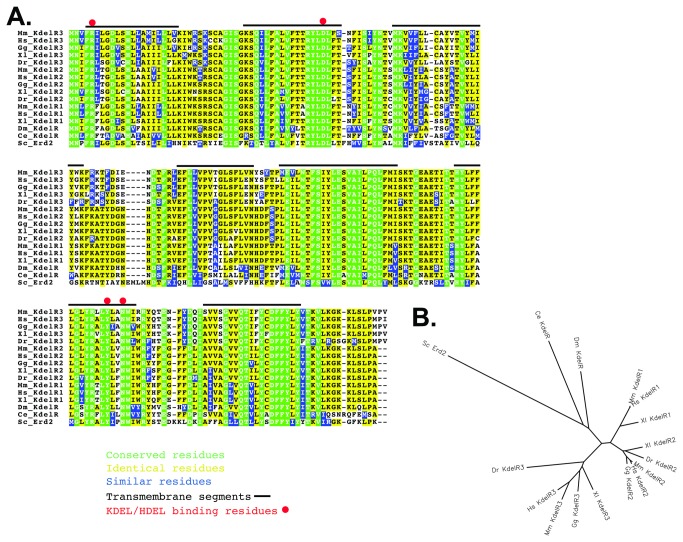
Drosophila KdelR is conserved. (A) Drosophila KdelR is highly conserved with respect to its vertebrate and *C. elegans* counterparts (A) and is homologous to *S. cerevisiae* Erd2. (Mm-mouse, Hs-human , Gg-chicken, Xl-*Xenopus*, Dr-zebrafish, Dm-Drosophila, Ce- C*. elegans* and Sc- *S. cerevisiae*). Vertebrates encode two-three Kdel Receptors, whereas only a single gene is found in flies, worms and yeast. Black bars over the sequences indicate membrane spanning regions [3]. Red dots denote residues involved in ligand (KDEL) binding [4]. (Green= completely conserved residues, yellow= identical residues, purple=similar residues). (B) A Phylip unrooted tree analysis of the KDEL Receptors from the major model organisms reveals that the Drosophila Kdel Receptor is slightly more related to the vertebrate proteins than are the worm and yeast receptors.

There is only a single Drosophila KDEL receptor gene, unlike in vertebrates where as many as three genes encoding highly related proteins have been discovered (Figure 1A,B; NCBI blast search) [24]. The ORF of the Drosophila KDEL receptor gene shows very high conservation with the vertebrate and *C. elegans* homologues and includes the seven membrane spanning domains as well as the residues that have been shown to interact directly with the KDEL motif of soluble ER resident proteins (Figure 1A, black lines and red dots) [3,4]. The Drosophila KDEL receptor shows somewhat less similarity to yeast Erd2p, but the position of the membrane spanning domains and the residues that interact with the HDEL/KDEL motif are conserved. 

### Identification of mutations in the *Drosophila* KDEL receptor gene

**Figure 2:**
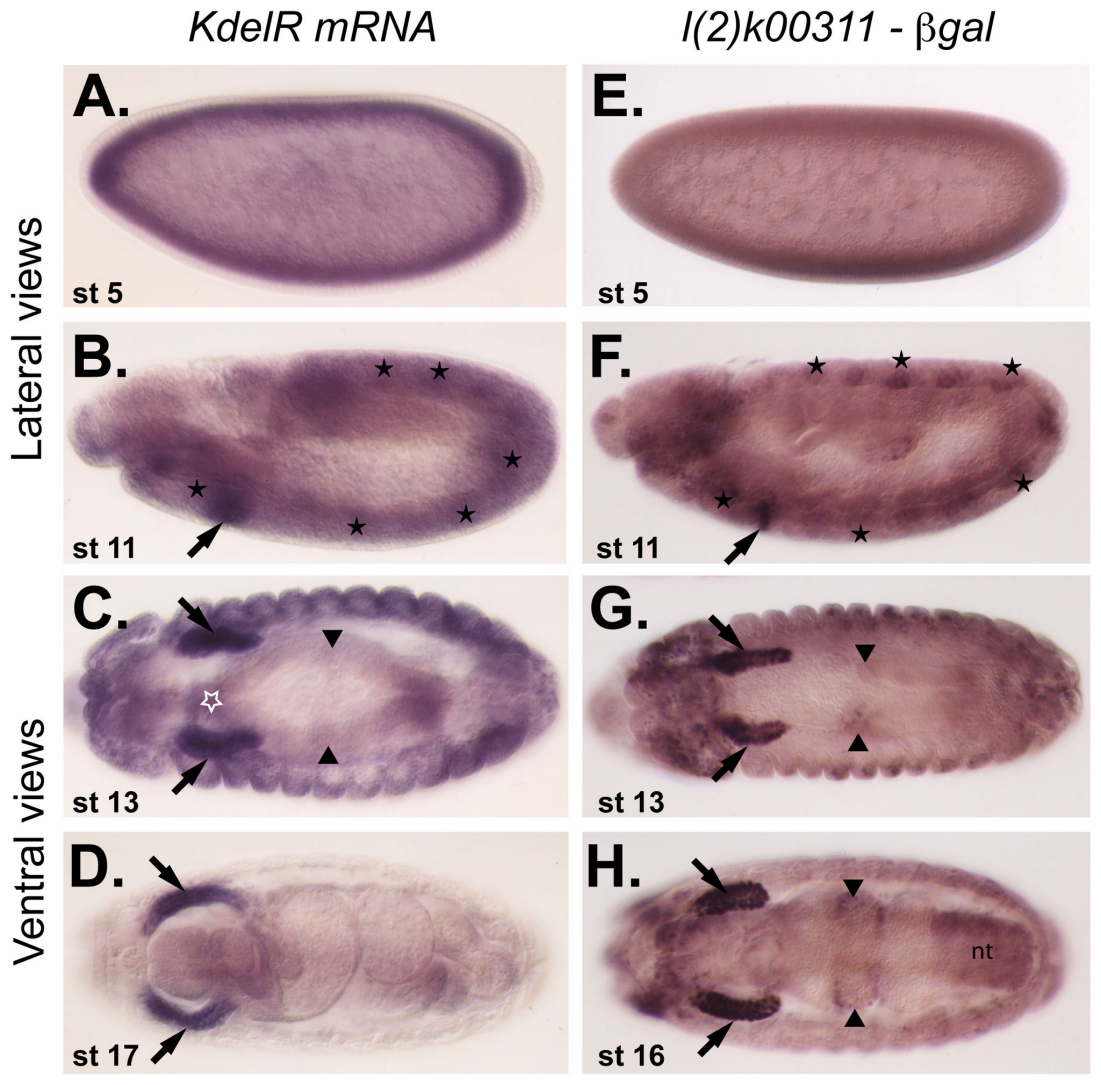
*KdelR* expression profile. *KdelR* is detected early in embryogenesis at the cellular blastoderm stage (A,E) and to high levels in the salivary gland beginning at embryonic stage 10 and continuing throughout embryogenesis (b-d, f-h, black arrows). *KdelR* is also expressed to elevated levels in the epidermis (cells on the embryo surface), fat body (clusters of staining in each segment in stage 11 embryos, indicated by black stars), proventriculus (white star) and a subset of gut endoderm cells (arrowheads). b-gal and RNA staining in the neural tube (nt) is also observed at late stages, although the RNA expression in the nt is not in the plane of focus of the embryos shown. Left column shows mRNA expression detected with a probe made from the *KdelR* cDNA clone CK00230 and the right column shows β-gal staining of *l*(*2*)*k00311*embryos.

**Figure 3:**
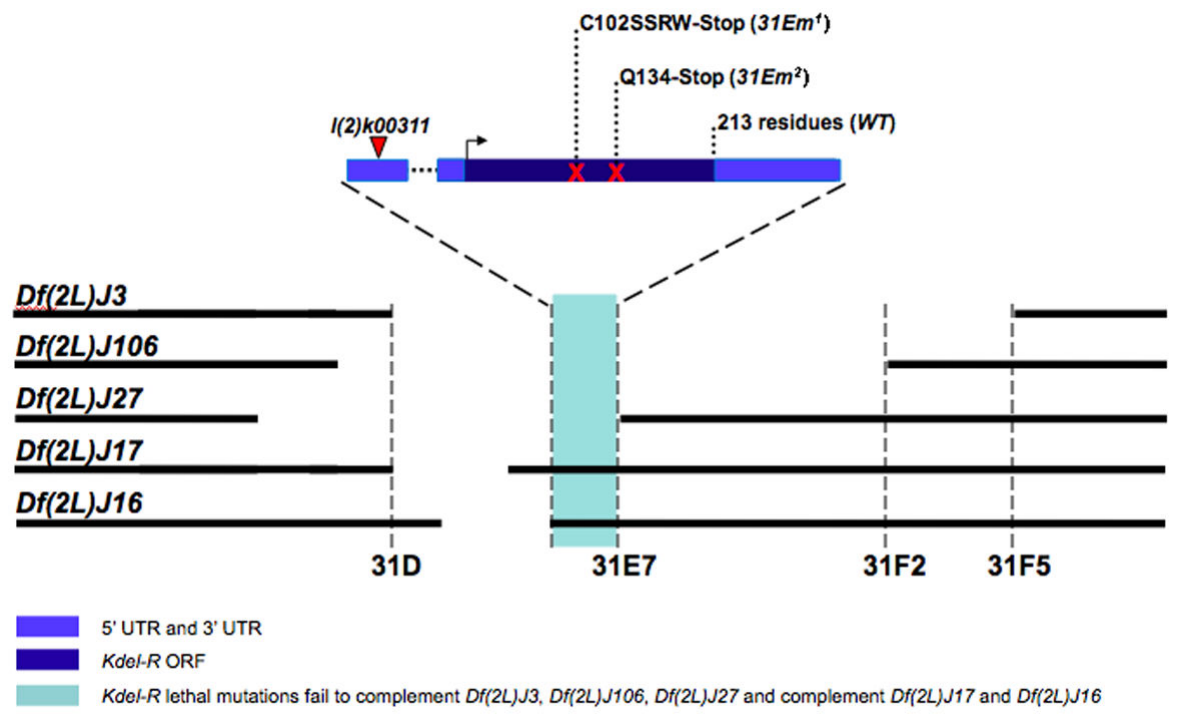
Identification of *KdelR* alleles. *KdelR* maps to region 31E in the Drosophila genome and *l*(*2*)*k00311* is inserted in the 5’ UTR of the *KdelR* transcript. *l*(*2*)*k00311* fails to complement deficiencies *Df*(*2L*)*J3, Df*(*2L*)*J106* and *Df*(*2L*)*J27*, but complements deficiencies *Df*(*2L*)*J17* and *Df*(*2L*)*J16*. Both EMS alleles of *KdelR* (*31Em1* and *31Em* ^*2*^) encode ORFs with premature stop codons.

 To gain genetic access to *KdelR*, we obtained a lethal P-element line, *l(2*)*k00311*, in which the P-element had inserted into the 5’ untranslated region (UTR) of the *KdelR* transcription unit. The pattern of -gal expression from the *l(2*)*k00311* line was very similar to that of the *KdelR* mRNA (Figure 2), with highest levels of expression in the secretory cells of the salivary gland. Elevated expression was also observed in the fat body (Figure 2B,F, stars), gut endoderm (Figure 2C,G,H, arrowheads), and epidermis. Both *KdelR* mRNA and -gal from the *l(2*)*k00311* insertion were observed in early (0-2 hr) embryos (Figure 2A,E), indicating maternal contribution. To identify additional *KdelR* mutations, we mapped the *l(2*)*k00311* lethality to a relatively small interval within cytological region 31E through complementation tests with several deficiencies in the genomic region to which *KdelR* localizes (Figure 3; [18]). *l(2*)*k00311* failed to complement *Df*(*2L*)*J3*, *Df*(*2L*)*J106* and *Df*(*2L*)*J27*, but complemented *Df*(*2L*)*J17* and *Df*(*2L*)*J16*. Six genes were known to map to the interval defined by these deletions [18] and both alleles of one of the six genes, *l(2*)*31Em*, failed to complement the lethality of *l(2*)*k00311*. Sequence analysis of the *KdelR* ORF of the *l(2*)*31Em* mutants revealed premature stop codons in each case (Figure 3). The resulting truncated proteins would be missing two of the predicted transmembrane spanning regions as well as two critical ligand interacting residues, indicating that *l(2*)*31Em* ^*1*^ and *l(2*)*31Em* ^*2*^ are likely null for *KdelR* function. 

### Lethal phase of *KDELR* mutants

 Erd2 is not essential for viability in yeast, but is required for growth [25]. In contrast, the *KdelR* is essential for viability of adult Drosophila. To pinpoint the stage of lethality, we assayed the survival of larvae at different stages of development. To identify mutants, we examined *l(2*)*k00311* larvae in a *yellow* (*y* ^*-*^) null background, balanced with a *CyO*, *y* ^+^ second chromosome. Mutant larvae, which are *y* ^-^ and have light brown mouthparts, can easily be distinguished from the wild-type larvae, which are *y* ^+^ and have black mouthparts. Overnight collections were aged for 24 hours (first instar) and 48 hours (second instar), and larvae were examined under a stereomicroscope. Examination of first instar larvae revealed actively moving wild-type larvae and very sluggish mutant larvae (data not shown). Second instar collections consisted of only live wild-type larvae (data not shown). Therefore, lethality occurs during the first instar larval stage. 

### *KDELR* is required for normal larval cuticle development

**Figure 4:**
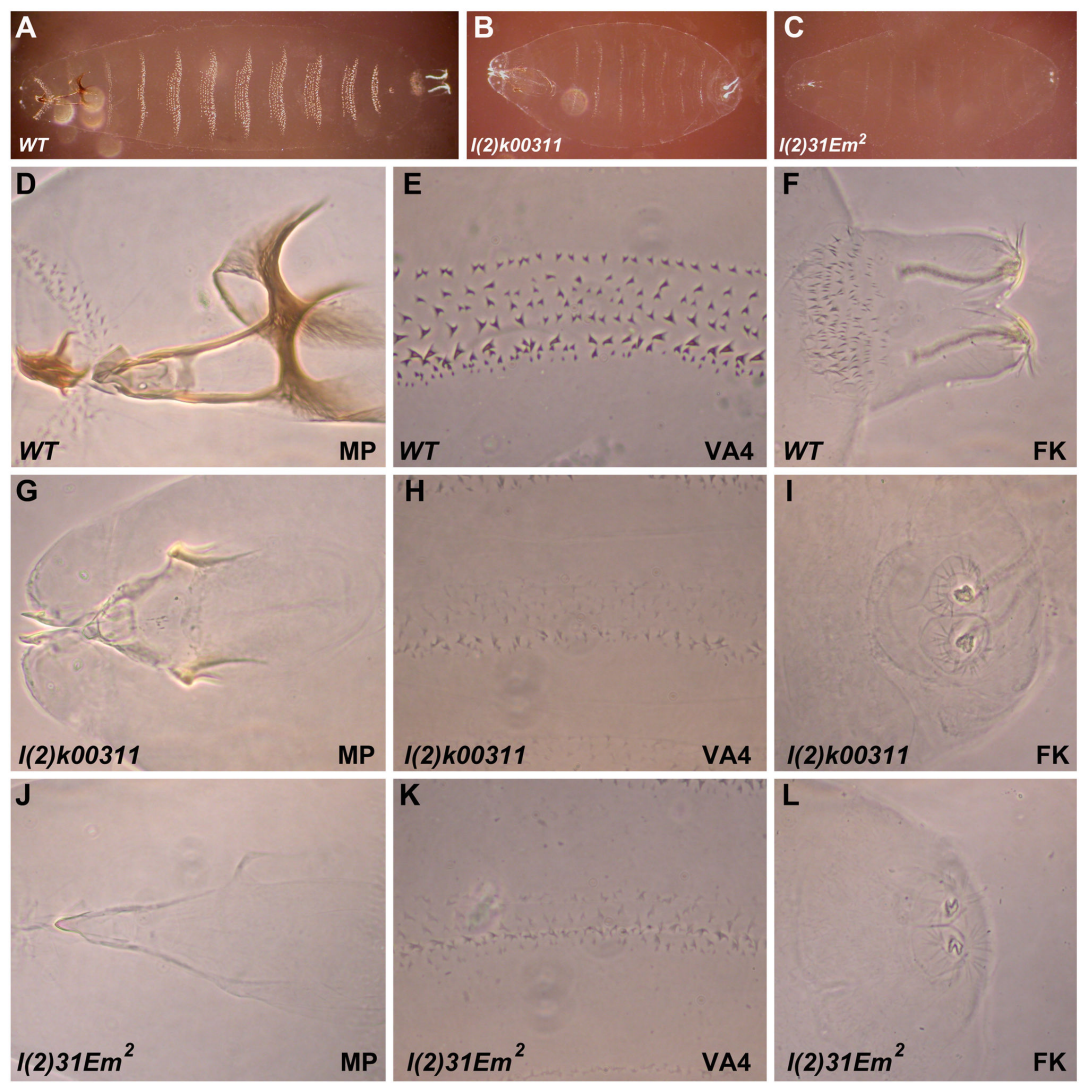
*KdelR* mutant cuticles are smaller than wild-type cuticles and are grossly underdeveloped. Dark field (ventral) images of wild-type, *l*(*2*)*k00311* and *31Em* ^*2*^ larvae (A-C). Note that *l*(*2*)*k00311* and *31Em* ^*2*^ mutants are approximately 60% the length of wild type larvae and the ventral denticles are not as prominent as in their wild-type siblings (A-C). Mouthparts (MP) of *l*(*2*)*k00311* (G) and *31Em* ^*2*^ (J) are underdeveloped and less pigmented than corresponding wild-type mouth parts (D). The filtzkörper (FK) of *l*(*2*)*k00311* (I) and *31Em* ^*2*^ (L) are underdeveloped and do not protrude from the larval body as in wild-type (F).

 Mutations that perturb normal secretory function in Drosophila have been shown to have profound effects on larval cuticle development [13,14,26]. Therefore, we examined the cuticles of *KdelR* mutants to ask if KDEL receptor loss-of-function affects cuticle development. *KdelR* mutant cuticles are much smaller and fainter than the corresponding wild-type cuticles (Figure 4A-C). The faint cuticle is most obvious when comparing the ventral denticle belts (Figure 4A-C, E, H, K). In addition, the mouthparts and filzkörper (tracheal filters) of *l(2*)*k00311* (Figure 4G,I) and *l(2*)*31Em* ^*2*^ (Figure 4J,L) are significantly underdeveloped compared to the corresponding wild-type structures (Figure 4D, F). These characteristics are consistent with the mutant defects seen with loss of other components of the secretory pathway as well as with loss of *CrebA*, which transcriptionally upregulates all known secretory component genes in specialized secretory tissues [13,14,26]. Thus, *KdelR* is essential for normal cuticle development.

### The KDEL receptor is required to maintain localization of some, but not all, ER resident proteins

**Figure 5:**
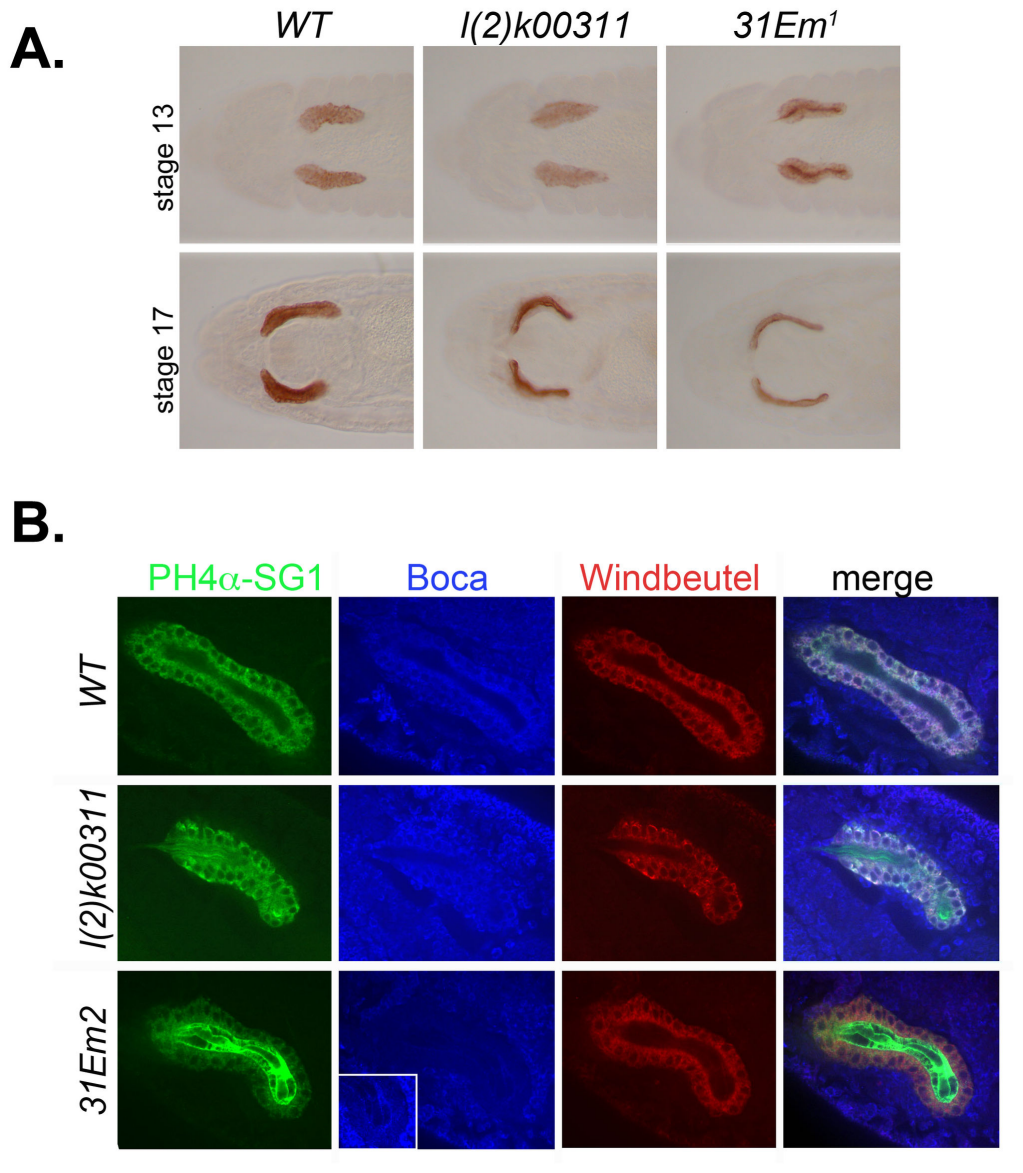
*KdelR* and the retention of PH4αSG1 and other soluble resident proteins in the ER. (A) Staining with antibodies to the resident ER protein PH4αSG1 revealed cellular expression in WT SGs beginning at embryonic stage 11 and continuing through embryogenesis (data not shown; left panels). By embryonic stage 13, PH4αSG1 staining was observed at low levels in the salivary lumens of *l*(*2*)*k00311* mutants (middle top panel) and at high levels in the salivary lumens of *l*(*2*)*31Em* ^*1*^ and *l*(*2*)*31Em* ^*2*^ mutants (top right panel and data not shown). By stage 17, almost all detectable PH4αSG1 was lumenal in both *l*(*2*)*k00311* and *l*(*2*)*31Em* ^*1*^ salivary glands. The same staining patterns were observed in all embryos examined of each genotype. (B) Stage 15 embryonic salivary glands were co-stained with antibodies to PH4αSG1, Boca, and Windbeutel. PH4αSG1 protein was observed entirely in the ER in wild-type salivary glands, in both the ER and lumen in *l*(*2*)*k00311* salivary glands, and predominantly in the lumen in *31Em* ^*2*^ mutant salivary glands (left panels). Boca protein was barely detected in the salivary glands of *31Em* ^*2*^ mutants, compared to the WT and *l*(*2*)*k00311* mutants, although some Boca protein can be detected in the lumens of *31Em* ^*2*^ mutants when the image is overexposed (second column, last row, inset). Wbl localization was largely unaffected in *l*(*2*)*k00311* and *31Em* ^*2*^ mutant salivary glands; only minimal lumenal staining of Wbl protein was observed, even when the cellular staining was at high levels (third column, second row). The changes in PH4αSG1 and Boca protein localization are more apparent in the merged images (last column). Again, the same patterns of accumulation were observed in all of the stage 15 embryos examined for each genotype.

To assay for *KdelR* activity, we compared the localization of an ER resident protein, PH4αSG1, in wild-type and *KdelR* mutant embryonic salivary glands. PH4αSG1 is highly expressed in the embryonic salivary glands [27] and encodes a putative prolyl 4-hydroxylase α (PH4α) subunit. PH4α-subunits are typically found in a complex with a corresponding KDEL containing β-subunit, which is thought to maintain the enzyme complex in the ER through its C-terminal KDEL motif [28,29]. PH4αSG1 is an ER resident based on its colocalization with the ER marker α-KDEL as well as with other GFP-tagged ER proteins ([21] RM Fox and DJA, unpubl.). α-PH4αSG1 staining, using a horseradish peroxidase (HRP) conjugated system, revealed changes in the cellular localization of PH4αSG1 in wild-type versus *KdelR* mutants. Whereas PH4αSG1 staining of heterozygous (wild-type) salivary glands revealed the reticular cytoplasmic localization characteristic of ER staining at all embryonic stages (Figure 5A, left panels), staining of *KdelR* mutants showed variable levels of staining in the salivary gland lumen. At earlier embryonic stages (stage 13), PH4αSG1 was detected in both the ER and lumen, although lumenal staining was relatively higher with the EMS null alleles (Figure 5A, top right panel and data not shown) than with the *l(2*)*k00311* mutant (Figure 5A, top middle panel), presumably because of residual expression from the intact *KdelR* ORF downstream of the insertion site of the P-element. At later stages, PH4αSG1 was nearly entirely lumenal (Figure 5A, bottom center and right panels), suggesting that maternally-provided *KdelR* mRNAs may partially rescue earlier ER retrieval function.

We then asked if *KdelR* function is required to maintain ER localization of other known soluble ER proteins containing C-terminal KDEL or KDEL-related motifs. Two such proteins include Boca, which is expressed in all cells and functions as a chaperone dedicated to the folding of low density lipoprotein receptors (LDLR) containing coupled -propeller/EGF modules [22,30], and Windbeutel (Wbl), which is a protein disulfide isomerase (PDI)-related chaperone expressed to very high levels in the embryonic salivary gland and required for folding of Pipe, a heparan sulfate 2-sulfotransferase [23,31]. We focused on stage 15 embryos to reduce the effect of potential maternal contributions and to optimize phenotypic differences between the null *l(2*)*31Em* ^*2*^ allele and the weaker *l(2*)*k00311* insertion allele. Indeed, a significantly higher level of α-PH4αSG1 was detected in the lumen of *l(2*)*31Em* ^*2*^ versus *l(2*)*k00311* salivary glands (Figure 5B, panels in column 1, rows 2 and 3). Furthermore, only low levels of α-PH4αSG1 were detected in the salivary gland cells of the *l(2*)*31Em* ^*2*^ mutants (Figure 5B, column 1, row 3 panel). Interestingly, although Boca protein was greatly reduced in the salivary gland cells in *l(2*)31Em^2^, it was barely detectable in the lumen (Figure 5B; overexposed inset in column 2, row 3 panel). The very low level of lumenal Boca staining may be due to epitope inaccessiblity in the lumenal environment. In any case, *l(2*)*31Em* ^*2*^ had a significant effect on Boca subcellular localization. Surprisingly, localization of the KEEL-containing protein, Wbl, was relatively unaffected in both *l(2*)*k00311* and l *( 2*)*31Em* ^*2*^ salivary glands (Figure 5B, panels in column 3), suggesting that Wbl ER retention is less dependent on *KdelR*. 

### The KDEL/KEEL sequence of Wbl and Boca are required for ER retention in S2 cells

**Figure 6:**
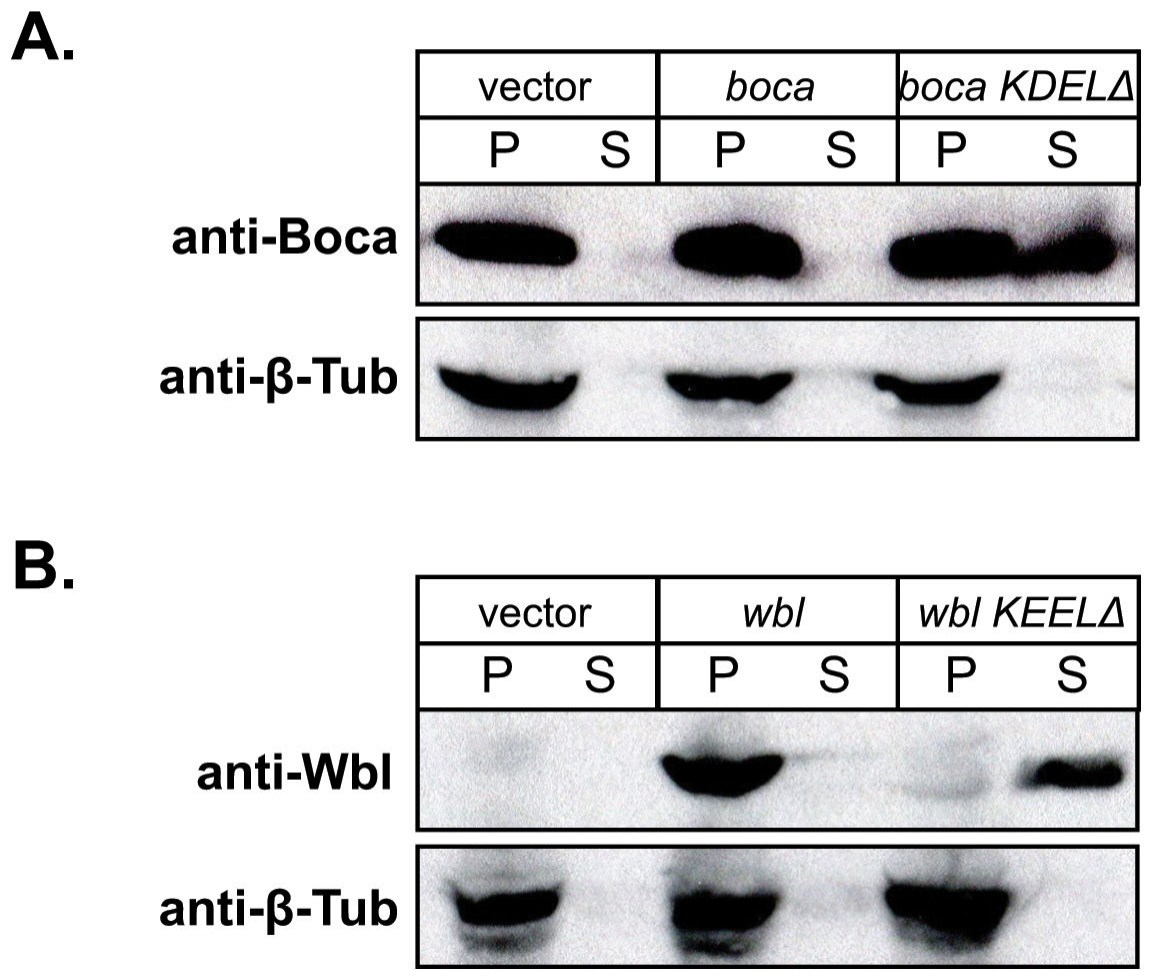
Boca and Wbl require C-terminal KDEL/KEEL sequences for ER retention in S2 cells (A) Immunoblots of cell pellets (P) and supernatants (S) from Drosophila S2 cells transfected with empty vector (left lanes), full length Boca (center lanes) or KDEL deleted Boca (right lanes) incubated with aBoca (top gel) or abTub antibodies (bottom gel). Note that Boca protein is easily detected in the cell pellets from all three samples but that Boca protein is also detected in the supernatant in only cells transfected with the KDEL-deleted Boca construct. Note also that bTub is detected in only the cell pellets from each transfected cell type, as expected for a cytosolic protein. (B) Immunoblots of cell pellets (P) and supernatants (S) from Drosophila S2 cells transfected with empty vector (left lanes), full length Wbl (center lanes) or KEEL-deleted Wbl (right lanes) incubated with aWbl (top gel) or abTub antibodies (bottom gel). Note that Wbl protein is detected in the cell pellets from S2 cells transfected with the full length Wbl construct, whereas Wbl protein is detected in the supernatant from S2 cells transfected with the KEEL-deleted Wbl construct. Note also that bTub is again detected in only the cell pellets from each transfected cell type.

To address the unexpected localization of Boca and Wbl observed in *KdelR* mutant salivary glands, we expressed either WT or KDEL/KEEL deleted versions of both proteins in tissue culture cells. Based on immunohistochemical staining, approximately 10 - 20% of S2 cells transfected with the Boca construct expressed very high levels of Boca protein; however, even with cells transfected with empty vector, we observed significant levels of Boca protein in all cells (data not shown), which is not surprising since Boca is ubiquitously expressed at all developmental stages that have been examined [22]. On the other hand, we did not observe Wbl in S2 cells tranfected with empty vector but did observe high level staining in approximately 10 - 20% of cells transfected with the Wbl constructs (data not shown), consistent with the more limited expression of Wbl in ventral ovarian follicle cells and embryonic salivary glands [23]. As expected, with cells transfected with constructs expressing WT versions of either Boca or Wbl, both proteins were found exclusively in the cell pellet (Figure 6A,B, middle sets of lanes), consistent with ER localization. On the other hand, with cells transfected with a mutant version of *Wbl*, which would encode a protein missing only the C-terminal KEEL sequence, all of the protein was found in the supernatant (Figure 6B, right set of lanes). Similarly, with cells transfected with a mutant version of *Boca*, which would encode a protein missing only the C-terminal KDEL sequence, a significant amount of protein was found in the supernatant, although the bulk of the protein (presumably from endogenous WT Boca expressed in every cell) was in the cell pellet (Figure 6A, right set of lanes). Thus, in S2 cells, ER retention of both proteins requires the cis-acting C-terminal KDEL/KEEL sequences. 

## Discussion

### Mutation in *KDELR* does not equally affect the localization of all ER resident proteins

To determine the utility of the Drosophila salivary gland as a system for studying mutations in secretory pathway components, we focused on *KdelR*, which encodes the only Drosophila KDEL receptor. We showed that loss of the receptor results in the depletion of the ER stores of some, but not all, soluble ER residents (Figure 5). Specifically, cellular levels of both PH4αSG1 and Boca were significantly diminished in late stage *KdelR* mutant embryos. As the PH4αSG1 protein levels declined in the ER, high levels were observed in the salivary gland lumen, consistent with the protein escaping to later secretory compartments and ultimately being secreted. Although Boca was clearly depleted in the ER of *KdelR* mutant salivary glands, corresponding increases in lumenal levels of Boca were not as obvious as with PH4αSG1. Nonetheless, we did observe that a mutant version of Boca, which deletes the KDEL sequence to which the KdelR binds, was easily detected in the supernatant of S2 cells, whereas WT Boca was not. Thus, the ER localization of two ER residents, PH4αSG1 and Boca, depend on a functional KDEL receptor. 

Interestingly, unlike PH4αSG1 and Boca, the Wbl protein appeared refractory to the loss of *KdelR* function in the salivary gland, suggesting that it is somehow maintained in the ER by other mechanisms. Nonetheless, as with mutant Boca protein, mutant Wbl, with a deletion of the C-terminal KEEL sequence to which the KdelR would presumably bind, was secreted into the cell supernatant when expressed in S2 cells, whereas a WT version of Wbl was not. This finding suggests that ER retention of Wbl in the salivary gland is mediated by factors in addition to the KdelR. Wbl could be maintained in the ER through its association with transmembrane ER residents, which are brought back to the ER in COPI vesicles. The COPI proteins interact directly with the dilysine motifs (KKXX) found at the C-terminus of transmembrane ER residents [32–34]. Alternatively, Wbl may be retained in the ER by interacting with calcium binding proteins such as calreticulin. Calreticulin, which also contains a KDEL sequence (HDEL in Drosophila; [12]), is still maintained in the ER even when expressed to levels that saturate the KDEL receptor function. However, when the calcium-binding domain of calreticulin is deleted, the calreticulin readily escapes [35]. 

Wbl could also be maintained in the ER through its association with salivary gland transmembrane ER proteins that are retrieved by the RER1 protein. RER1 has been shown to be a retrieval receptor for ER membrane proteins and functions independently of both the KDEL signal and the dilysine signal systems [36]. A clear Drosophila homologue to RER1 exists in Drosophila (CG11857) and is also transcriptionally upregulated in the salivary gland (http://www.fruitfly.org/EST/index.shtml). Thus, the Drosophila salivary gland may utilize much of the diverse cellular machinery for the maintenance of ER proteins and that machinery may be differentially employed depending on the ER resident in question. It should be pointed out, however, that in yeast, an independent system is used to maintain levels of ER proteins in *erd2* mutants [37]. This system is activated by the IRE1 endonuclease, which splices the *HAC1* transcript into a form encoding an active transcription factor in response to stress, such as the loss of *Erd2* function [38]. Although more ER residents escape, more are quickly introduced into the system by increased transcriptional activation of ER protein genes. An IRE1 homologue exists in Drosophila and could contribute to a failure to see significant reduction in the levels of Wbl in the *KdelR* null embryos. In this scenario, increased levels of Wbl, like Boca, may not be detectable in the lumen due to problems with epitope accessibility or antigen recognition in the lumenal environment. 

### Larval cuticle defects can reveal novel secretory components

 Here, we show that loss of function mutations in *KdelR* result in severe cuticle defects, including a shortened, underdeveloped cuticle, wherein the ventral denticles, mouthparts and filzkörper are barely visible. Similar phenotypes have been observed with P-element insertion mutations in several other genes known to encode core secretory machinery as well as with null mutations in the *CrebA* transcription factor gene, which is known to coordinately upregulate secretory machinery genes in specialized secretory cells – including the epidermal cells that secrete the larval cuticle [13]. The consistency in cuticle phenotypes observed with known secretory pathway mutants and the easy (inexpensive) protocols for preparing cuticles for microscopic examination make this an ideal system for screening for novel essential secretory pathway genes – simply assay for weakened cuticles without changes in denticle patterning.

### The salivary gland as a model system for studying secretion

 This study supports the use of the Drosophila salivary gland for studying secretory pathways components *in vivo*, in the context of an organ system specialized for secretion. The salivary gland is a monolayered epithelial tube comprised of large polarized cells in which it is easy to visualize organelles and subcellular compartments. We have reported elsewhere that the genes encoding the machinery known to be required at early steps in the secretory pathway are expressed to very high levels relatively early in the salivary gland [13]. This tissue will persist for several more days without undergoing cell division, a process that might otherwise complicate studies of organelle maintenance and function. The maternal contribution of the secretory genes, such as *KdelR*, is likely to allow development to proceed to a stage where the salivary glands have formed and are functional. The effects of zygotic loss of the secretory genes can then be studied as the maternal supplies are depleted. A survey of Flybase (http://flybase.bio.indiana.edu) indicates that mutations exist for at least one subunit of each of the known complexes involved in early steps in the secretory pathway. Hence, the starting materials are readily available for testing the proposed roles of the known secretory components in a eukaryotic organ specialized for secretion. Indeed, our identification of null mutations in the single Drosophila *KdelR* will allow for more direct tests for a role for the KdelR in inducing autophagy in neurodegenerative disease models [39] and in regulating cargo flux through the Golgi [40]. The null mutations can also be used to clarify the importance of post-translational modifications on Kdel-R localization and activity [41]. 
